# Performance Studies of Aluminium-Based Gas Electron Multiplier Detector

**DOI:** 10.3390/s24134169

**Published:** 2024-06-27

**Authors:** Bartosz Mindur, Tomasz Fiutowski, Piotr Wiącek

**Affiliations:** AGH University of Krakow, Faculty of Physics and Applied Computer Science, al. A. Mickiewicza 30, 30-059 Krakow, Poland

**Keywords:** micropattern gaseous detectors, gaseous imaging and tracking sensors, X-ray sensors

## Abstract

In this paper, we report on a systematic study of a soft X-ray Gas Electron Multiplier (GEM) detector built with aluminium-clad kapton GEM foils. The primary objective of this research is to comprehend the performance of this type of detector when irradiated with soft energy photons. The results are analysed and discussed with a particular focus on the long-term detector stability, as well as its gas gain and energy resolution uniformity across the detector area. Presented results lead us to the conclusion that the aluminium based GEM detector is a promising device to suppress the X-ray Fluorescence (XRF) background, simultaneously providing very good stability during long-term measurement campaigns.

## 1. Introduction

Among the various Micro-Pattern Gaseous Detectors (MPGDs), Gas Electron Multipliers (GEMs) are the most common and widely used in various research areas [1,2,3,4]. They are relatively easy to assemble from prefabricated components and to configure in order to adapt their parameters to specific requirements [5,6,7,8,9,10,11,12,13,14,15]. The GEMs especially find application in projects where high stability, high counting rate, and good spatial resolution are required. They are also now widely used to measure soft X-rays [16,17,18], despite their rather moderate energy resolution [19,20].

A standard GEM detector is made up of copper-rich components, which in the case of soft X-ray detection can be quite a problem. When the detector is irradiated with primary photons of sufficiently high energy (above the copper absorption edge), the copper fluorescence X-rays produce a significant background signal, which introduces unwanted distortion into the analysis. To overcome this problem (already reported previously), we have proposed a post-processing step for standard copper-clad GEM foils to remove the copper cladding from most of the foil area and retain only the adhesive chromium layer. Our previous measurements confirmed that the use of such foils helps significantly suppress the copper-originating fluorescence background, but does not completely remove it [10,21]. Eventually, the elimination of the background signal would be possible if copper is completely removed from the structural components of the detector and the GEM foils themselves are made of much lighter elements, such as aluminium. In recent years, it has finally been possible to produce films with the desired characteristics and to build aluminium-clad GEM detectors [22,23].

In this paper, we present the results of more than six months of measurements demonstrating the long-term stability of the basic parameters of the aluminium-clad GEM detector, i.e., gas gain and energy resolution. Based on these results, it can be concluded that the performance of the detector has not deteriorated during that time and that type of the GEM detector can be used in experiments requiring operation for extended periods without the necessity to apply additional correction factors.

## 2. Materials and Methods

### 2.1. Aluminium-Based GEM Detectors

**Figure 1:**
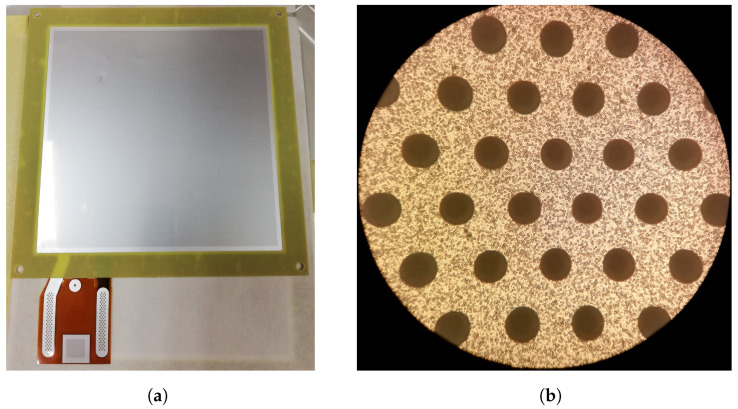
Photographs of the aluminium GEM foil. (**a**) View of the whole 10 cm × 10 cm foil (framed—ready to mount in the detector). (**b**) Magnified microphotography of the foil.

The detector used in this study is similar to the standard triple-GEM detector, which has been produced for many years using copper-clad foils [5]. The manufacturing process was carried out at the European Organization for Nuclear Research (CERN) by the Micro-Pattern Technologies (MPT) laboratory [24]. The MPT laboratory developed a specially optimised process and prepared the aluminium coating kapton drift and GEM foils (photographs of an aluminium GEM foil are presented in Figure 1).

Aluminium coating is produced through vacuum deposition using a sputtering method. Initially, a deposited aluminium layer is much thicker than desired and is subsequently reduced to 5 μm by chemical etching with soft phosphoric acid. In contrast to traditional copper-clad GEM foils, the aluminium coating does not have the chromium substrate that is usually found between the kapton and the copper [23]. This design choice eliminates not only the potential source of unwanted background radiation from copper but also from heavier elements with significant X-ray fluorescence photon energy. The necessity of having low-Z materials in detector construction is crucial, as already reported in [10,11,12,16,25].

**Figure 2:**
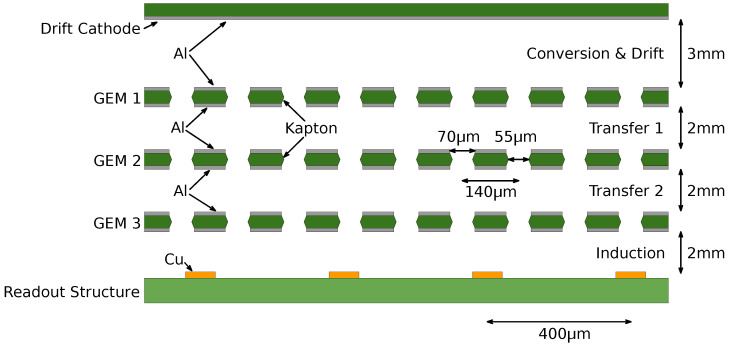
Schematic diagram of the GEM detector. It has to be underlined that most of the detector components are made solely with low-Z elements.

All other detector components are similar to those used for the standard GEM. A schematic diagram of the detector’s inner components is shown in Figure 2. The detector is assembled in a gas-tight chassis, containing a low-mass kapton window, aluminium drift electrode, three aluminium GEM foils, and Cartesian readout structure with two sets of orthogonal strips in a pitch of 400 μm. The GEM detector has an active area of 10 cm × 10 cm in the form of a square entrance window. The drift region (volume between a drift foil and the first GEM foil) has a thickness of 3 mm, and all other regions (transfer and induction) have a thickness of 2 mm. This construction of the GEM detector is exactly the same as the ones used in our previous studies [21,25] so the results between different detector assemblies can be directly compared. The detector preparation procedure before a measurement campaign was also very similar to that established for earlier works. All detector components were thoroughly cleaned and carefully mounted into the GEM detector in a dedicated clean-room environment at AGH University of Krakow.

### 2.2. Detector Test Stand

A specially engineered testing configuration, designed for both spectroscopic and imaging studies, was employed to methodically evaluate the detectors’ performance. Inside our detector laboratory, all tests were conducted under controlled temperature conditions using an air conditioning system. The temperature remained within a range of about ±2 °C. The gas pressure followed the atmospheric pressure with a slight over-pressure. In addition, the gas flow rate was maintained at a level of at least two detector volume exchanges per hour. Parameters and characteristics of the tested GEM detector were evaluated based on the measured spectra of ^55^Fe radioactive source (photon energy equal to 5.90 keV). The radioactive source was positioned above the detector entrance window at a distance of ~20 cm in order to keep non-uniformity of the photon intensity over the detector below a factor of two.

During the studies, a dedicated readout system for a GEM detector equipped with a custom-made Application Specific Integrated Circuits (ASICs) and Field-Programmable Gate Array (FPGA) was used. More about the GEMROC ASIC, Data Acquisition (DAQ) system and software can be found in [26,27]. All the measurement equipment was the same as in our previously published studies in [21].

### 2.3. Measurement Procedure

The recently constructed aluminium GEM detector underwent a week-long process of biasing and flushing with an Ar/CO_2_ 70/30 gas mixture before any measurements were performed. The Ar/CO_2_ 70/30 is a standard gas mixture used in GEM detector characterization and it was also used in our previous measurements reported earlier. Subsequently, systematic studies were carried out, with particular attention paid to the detector’s long-term performance. Additionally, periodic tests were performed at varying high-voltage bias settings.

## 3. Results and Discussion

In this section, we present the results of the measurements of the GEM detector with aluminium-clad foils. These results are based on measurements taken with a readout pitch of 800 μm, i.e., for pairs of neighbouring strips connected to individual channels of the front-end electronics. This scheme results in 128 readout channels for each coordinate and 128 × 128 pixels for the entire detector, each of 800 μm × 800 μm [26].

There are significant advantages in using foils with no heavy elements, which results in no background fluorescence radiation (from copper and chromium excitation). These advantages have been extensively discussed in the context of soft X-ray photon detection (e.g., in [10]). The actual measurements were performed in the same manner as in our previous studies [21,25]. Therefore, the results presented below can be directly compared to former measurement outcomes.

First, we verify the overall detector performance by creating the gas gain and energy resolution maps across the detector area. Then, we present the results of the detector bias voltage scan. Finally, we show the results of the long-term stability test.

### 3.1. Gas Gain and Energy Resolution over the Detector

**Figure 3:**
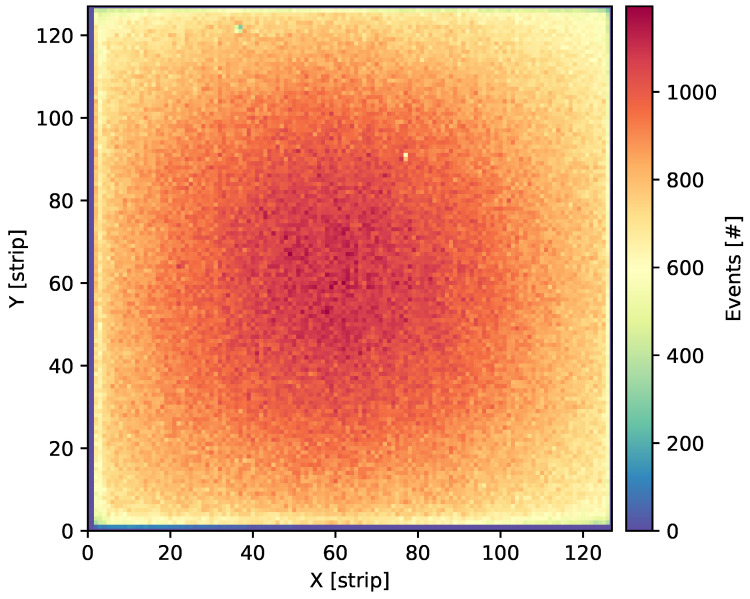
The ^55^Fe irradiation map for the aluminium GEM detector is presented in the form of a cumulative number of counts over the whole active area. Measurements were performed at a bias voltage of 3880 V and the recorded average rate of photons was equal to 30.8 cps/mm^2^.

The first verification step is to build an ^55^Fe irradiation map as a form of the cumulative counts over the whole detector active area. The reconstruction result is plotted as shown in Figure 3.

The cumulative count map provides a rapid, qualitative assessment of the detector’s performance. As expected, since the source is located about 20 cm above the detector surface, we can observe most events in the centre and a gradual decay towards the edges, as can be clearly seen in the map. Such a picture is a good indicator of the proper detector’s performance.

The next step is to create gas gain and energy resolution maps across the detector’s surface. To construct comprehensive maps of these parameters, an event reconstruction procedure is utilised to build individual energy spectra for all detector “pixels”. The reconstruction procedure is the same as described in our previous reports [21,25].

**Figure 4:**
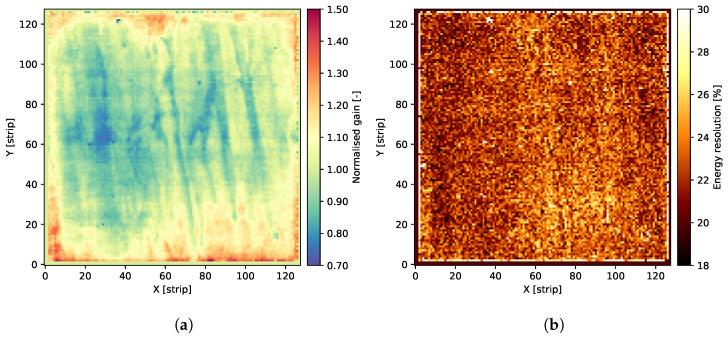
Maps showing gas gain and energy resolution distributions at the beginning of long-term studies of the GEM detector. (**a**) Normalised gas gain map over the detector area. (**b**) Energy resolution map over the detector.

**Figure 5:**
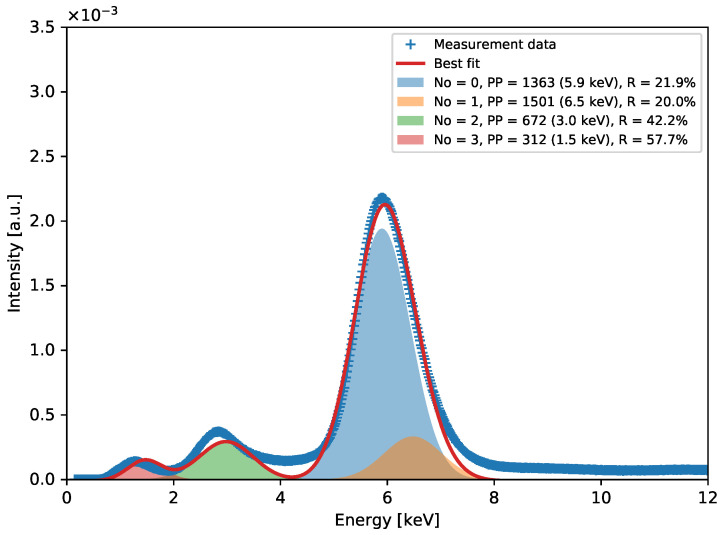
Spectrum for ^55^Fe source at 3880 V for GEM detector with aluminium-clad foils.

Individual spectra acquired using ^55^Fe radioactive source feature two distinct peaks corresponding to the Mn K*_α_*-line of 5.90 keV and the argon escape peak corresponding to an energy of 2.94 keV. The signal amplitude related to the main peak is utilised to estimate the gas gain. The data are fitted to a single Gaussian distribution, yielding the peak position and the standard deviation or Full Width at Half Maximum (FWHM). The FWHM value of the 5.90 keV photo-peak serves as a metric for the local energy resolution throughout the detector. However, it is important to note that the energy resolution derived from fitting the data to a single Gaussian peak is influenced by the presence of the Mn K*_β_*-line of 6.49 keV. Given that the statistics of recorded events for individual “pixels” are relatively low, fitting these data to a distribution consisting of two peaks is not considered reliable. In the end, the average value for the entire detector is calculated for the peak positions, and then the gas gain map is normalised to this average level, as shown in Figure 4a. Hence, the maps depicted in Figure 4b should be interpreted as visual representations of the energy resolution’s variability across the detector’s surface, rather than an assessment of the detector’s maximum energy resolution. A more general assessment of an overall energy resolution can be derived from the cumulative spectrum, as shown in Figure 5. The value of about 21.9% for the Mn K*_α_* fluorescence line is quite a typical energy resolution for a GEM detector.

After applying the gas gain normalisation, a cumulative spectral plot for all events recorded by the detector is finally created. A typical spectral plot for the ^55^Fe radioactive source, corresponding to the irradiation map presented in Figure 3, is shown in Figure 5. Apart from the main peak at 5.90 keV (with 6.49 keV) and the escape peak at 2.94 keV, the spectrum also features a small peak at 1.49 keV corresponding to the aluminium K*_α_* fluorescence line from the aluminium-clad GEM and drift foils.

### 3.2. Detector Bias Voltage Scan

**Figure 6:**
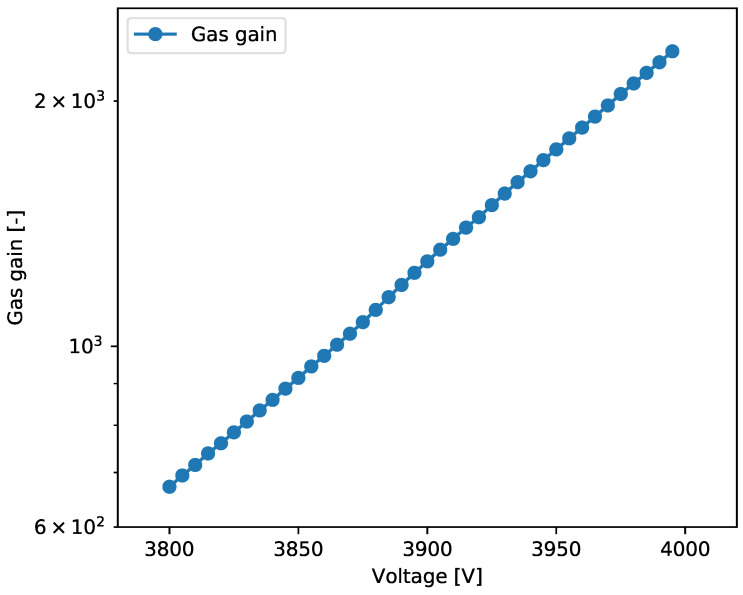
Gas gain as a function of bias voltage for GEM detector with aluminium-clad foils.

To investigate the performance of the detector, a few measurements were repeated at different detector bias voltages, resulting in a change in gas gain from approximately 600 to 2200. Figure 6 presents a typical plot of detector gas gain as a function of detector bias voltage.

As expected, the gas gain increases exponentially with a growth in the bias voltage. There are no significant deviations from the expected behaviour, which is a good indicator of the detector’s stability. The gas gain values are consistent with the results obtained in previous studies [21,25]. The actual values are slightly lower than those using chromium-clad foils and are comparable to standard copper-clad foils [21]. This is most probably due to the smaller overall thickness of the chromium-clad foils (reduced by etching of the copper layers), which results in a higher electric field inside the holes and thus higher gas gain.

### 3.3. Long-Term Stability

In the case of radiation detection, the overall system performance should be stable for an extended period without any extensive calibration actions needed. The stability and repeatability of the system performance are very crucial, especially for spectroscopic measurements, where determining the energy of the incoming radiation is a must. There are a variety of applications where long-term stability is a crucial feature, e.g., X-ray Fluorescence (XRF) imaging of art objects in the field of cultural heritage [12,28,29]. Therefore, we conducted a dedicated long-term measurement of the GEM detector with aluminium-clad foils to extensively evaluate this detector type. It should be underlined that continuous measurements are not a standard procedure for GEM detectors, which require a dedicated test stand and stable conditions for a long time. Our detector was continuously operating for about half a year.

The essence of the test procedure lies in the continuous sampling of the signals generated by photons emitted from the ^55^Fe radioactive source. Interestingly, despite the passage of time, most operational conditions remained relatively stable. However, subtle fluctuations in ambient temperature and atmospheric pressure were present. The laboratory air conditioning system did not account for these variations, especially during seasonal changes (from summer to winter). These minor changes do not significantly impact the overall accuracy of the results.

**Figure 7:**
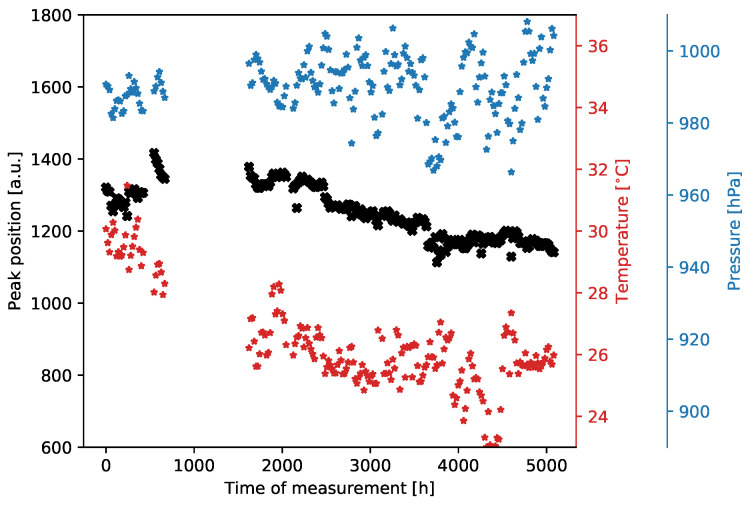
Long-term operation of the GEM detector with aluminium-clad foils. Black crosses represent the peak position, while red and blue stars represent the gas temperature and pressure, respectively. One can observe that the average gas gain (represented by the peak position) is kept almost constant, despite there being a season change and no (re-)calibration procedure applied.

**Figure 8:**
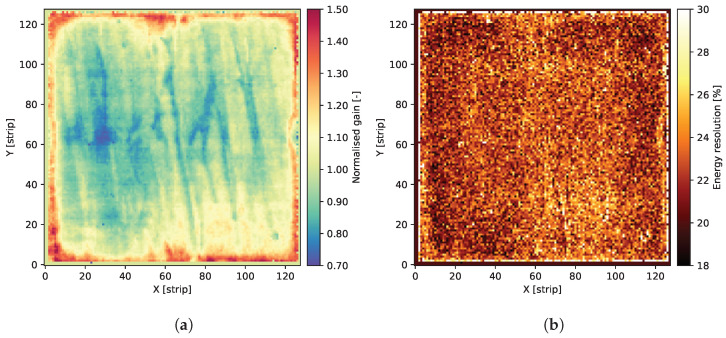
Maps showing gas gain and energy resolution distributions after almost half a year of continuous irradiation of the GEM detector. (**a**) Map of the gas gain over the detector area. (**b**) Map of the energy resolution over the detector area.

The overall result of the long-term operation is shown in Figure 7. As a result of the long-term measurement, one can observe that the gas gain, represented by the peak position of spectra collected across the entire detector, is kept almost constant. Some variations are most probably due to environmental changes in the laboratory, mostly ambient pressure and temperature. Therefore, we can conclude that the detector is operational stably and reliably. It is really important to note that the overall measurements took almost 5000 hours. Additionally, to verify the operation during the long-term measurements, the gas gain and energy resolution maps were periodically checked. Examples of resulting maps obtained after almost half a year of continuous irradiation are shown in Figure 8. There are also no significant changes in these maps compared to the beginning of the irradiation period, which is another indicator of the detector’s stable performance.

## 4. Conclusions

The GEM detector with aluminium-clad foils yields results consistent with the more traditional copper-clad foil design. The validity of previously used measurement and analysis methods, including the calibration procedure, gas gain and energy resolution determination, and long-term stability, is also demonstrated for this design. A comparison between the aluminium-based GEM detector and the traditional one (with copper-clad foils), utilising a standard method of registering X-ray photons from an ^55^Fe radioactive source, does not highlight significant differences between them. Long-term studies show that the detector performance is stable over time, which is a crucial aspect of the detector’s applications in a variety of experiments. The previously reported significant issue with the copper-clad foils’ extensive response to photons with energy exceeding the copper fluorescence line mostly disappears for the GEM detector with aluminium-clad foils, leaving only a slight influence on the lower-energy part of the spectrum (around 1.49 keV) by the low-yield aluminium fluorescence line.

Based on the results of the measurements that have been carried out, it can be concluded that GEM detectors built with aluminium-clad foils are promising candidate for detecting and analysing soft X-ray emissions, providing stable operation over an extended time. They can be particularly useful in applications such as XRF imaging and plasma diagnostics, where their greatest advantage lies in reducing unwanted background fluorescent radiation from copper, which compensates for the significantly higher cost of producing aluminium-clad foil due to a more complex technological process.

## Data Availability

Dataset available on request from the authors.

## Abbreviations

The following abbreviations are used in this manuscript:

ASIC	Application Specific Integrated Circuit
DAQ	Data Acquisition
FPGA	Field-Programmable Gate Array
FWHM	Full Width at Half Maximum
GEM	Gas Electron Multiplier
MPGD	Micro-Pattern Gaseous Detector
MPT	Micro-Pattern Technologies
XRF	X-ray Fluorescence
